# Measuring effective orifice area of bileaflet mechanical valves in patients after aortic valve replacement using phase-contrast cine MR imaging

**DOI:** 10.1186/1532-429X-13-S1-P325

**Published:** 2011-02-02

**Authors:** Haruhiko Machida, Masami Hirata, Shinya Kojima, Kazufumi Suzuki, Eiko Ueno, Akihito Sasaki, Kiyoharu Nakano

**Affiliations:** 1 Tokyo Women's Medical University Medical Center East, Tokyo, Japan

## Introduction

Mismatch of patients and prostheses is a critical issue for patients undergoing aortic valve replacement (AVR), because it increases postoperative mortality. Mismatch can be determined by measuring the effective orifice area (EOA) of a prosthetic valve. Although EOA is usually measured by transthoracic ultrasonography, its accuracy remains unclear because the prosthetic valve and/or turbulent flow may produce artifacts and results depend on the examiner’s skill.

## Purpose

We investigated the clinical feasibility of phase-contrast (PC) cine MR imaging for measuring EOA of bileaflet mechanical valves widely used in patients after AVR, which we believe has not been reported, and compared our measurements to EOA reference values and the differences between 2 different vendors.

## Methods

We retrospectively recruited consecutive 17 asymptomatic patients (8 men, 9 women; mean age, 66.2 ± 15.2 years) who underwent AVR using bileaflet mechanical valves of 2 different vendors, St. Jude Medical (SJM) (n = 7) and ATS (n = 10) valves, and demonstrated no significant abnormality on transthoracic ultrasonography. We first performed segmented k-space, PC cine MR imaging at the level of the aortic annulus (AA) and left ventricular outlet tract (LVOT), then measured velocity-time integral (VTI) during the systolic cardiac phase at both the AA and LVOT levels and functional cross-sectional area (A) at the LVOT level. We finally calculated EOA using the following continuity equation: EOA = A (LVOT) x VTI (LVOT) / VTI (AA). We used linear-regression analysis and Bland-Altman plot to compare the EOA measurement and EOA reference value for the type and size of prosthesis being implanted. We also used Student-t test to compare the difference between the EOA measurement and EOA reference value between the 2 different vendors.

## Results

Using PC cine MR imaging, we successfully obtained EOA measurement for all patients with little artifact from the prosthetic valve. The measurement and reference value correlated well (MR measurement = 0.91x + 0.24 cm^2^: r = 0.67, p = 0.002). All differences were within 0.2 cm^2^ except 3 measurements (difference, 0.3 to 0.7 cm^2^); the mean difference was 0.11 and the standard deviation, 0.25. There was no significant difference between those differences of SJM (0.23 ± 0.21 cm^2^) and ATS (0.02 ± 0.26 cm^2^) valves (p = 0.096).

## Conclusions

PC cine MR imaging is clinically feasible for relatively easy and accurate EOA measurement of bileaflet mechanical valves in patients after AVR.

